# Epidemiology of C2 Fractures in the 21st Century: A National Registry Cohort Study of 6,370 Patients from 1997 to 2014

**DOI:** 10.1155/2017/6516893

**Published:** 2017-10-17

**Authors:** Anna-Lena Robinson, Claes Olerud, Yohan Robinson

**Affiliations:** ^1^Stockholm Spine Center, Upplands Väsby, Sweden; ^2^Department of Surgical Sciences, Uppsala University Hospital, Uppsala, Sweden

## Abstract

**Objective:**

C2 fractures are a common injury in the elderly population. Treatment is often complicated due to osteoporosis and patient comorbidity. This study aims to investigate the incidence and treatment trend of C2 fractures in Sweden.

**Methods:**

Patients with the principal and secondary diagnosis of fracture of the second vertebrae (ICD-10: S12.1) between 1997 and 2014 were identified in the Swedish National Patient Registry (NPR).

**Results:**

Between 1997 and 2014, 6,370 patients with a C2 fracture (51% male; age: 72 ± 18) were identified in the NPR. The incidence of C2 fractures increased from 3 to 6 per 100,000 (*r* = 0.94; *p* < 0.01), mainly due to an increase of incidence in the geriatric subgroup (≥70 years). The percentage of surgically treated patients decreased from 1997 to 2014 (*r* = −0.80; *p* < 0.01). Younger age, male gender, spinal cord injury, and earlier year of admission were associated with surgical treatment assignment.

**Discussion:**

This study documents a rising incidence of C2 fractures in the elderly during the last two decades in Sweden. Greater awareness of fractures, improved diagnostics, coding, and a higher activity level of the patients are plausible causes. The declining trend of surgical treatment warrants further study.

## 1. Introduction

Fractures of the second cervical vertebra (C2) are a common injury in both the elderly and the young and active population [1]. Previously published studies find 9–18% of cervical fractures to be C2 fractures, of which 35–78% are odontoid process fractures and 11–25% are traumatic C2 spondylolysis, Hangman's fractures [2–6].

In the elderly, the proportion of C2 fractures is greater than that in the younger population [7, 8]. The elderly population has grown during the last decades [9]; therefore it is likely that the incidence of C2 fractures has increased as well. About 89% of the C2 fractures in patients ≥ 70 years of age in two tertiary referral centres in Sweden are odontoid fractures [8]. On a regional level in Sweden, we report a growing incidence of elderly patients with C2 fractures, which has not been seen in the younger population [8].

Surgical treatment options vary depending on the type of C2 fracture. Odontoid fractures type 2 are commonly treated with anterior screw osteosynthesis or posterior C1-C2 fusion [10–13]. Hangman's fractures are treated surgically with anterior C2-C3 fusion, posterior direct osteosynthesis, or posterior C2-C3 fusion [14, 15].

Nonsurgical treatment of C2 fractures is commonly performed with a rigid cervical collar [16]. In cases of instability or dislocations a halo-vest treatment is possible [17, 18].

The availability of prospectively collected data in nationwide registries in Sweden allows tracking of epidemiology retrospectively without the necessity of repeated cross-sectional trials. This study aims to investigate the incidence and treatment trend of C2 fractures during the last two decades in the Swedish National Patient Registry.

## 2. Patients and Methods

### 2.1. Study Design

This national multiregistry cohort study used prospectively collected electronic healthcare data from the Swedish National Patient Registry (NPR) and Statistics Sweden between 1997 and 2014. The study protocol was approved by the Institutional Ethical Review Board (2010/131/1) and follows STROBE and RECORD statements [19].

### 2.2. Setting

The Swedish National Patient Registry is hosted by the Swedish National Board of Health and Welfare and contains all patient contacts within Sweden with a coverage of >90% for orthopaedic diagnoses [20]. Registered are main diagnosis and comorbidity using the International Classification of Diseases, Ninth Revision (ICD-9), until December 1996 and since then the ICD-10 code [21]. In the International Classification of Diseases, Tenth Revision (ICD-10), there is no subclassification for C2 fractures [21]. Treatment has been coded since 1997 using the Swedish classification of surgical procedures [22]. Furthermore, information on hospitalisation time is available from the registry. Statistics Sweden is an administrative agency, providing statistics to the government, different agencies, and researchers.

### 2.3. Participants

All patients registered with the main and secondary diagnosis of C2 fracture treated between 1 January 1997 and 31 December 2014 were extracted from the NPR. In this study, we wanted to calculate the incidence and treatment trend, and therefore both main and secondary diagnoses were included, so that most possible C2 fractures would be included for calculations. Prior to data transmission, the Swedish National Board of Health and Welfare anonymised the individual personal identification numbers using a key that remained with the agency. Patients younger than 20 years of age and older than 99 years at the date of fracture were excluded. Population registry data from January 1997 until December 2014 were abstracted from Statistics Sweden. An inclusion flow diagram was prepared according to CONSORT statements [23].

### 2.4. Variables

The ICD-10 code S12.1 (fracture of the second vertebrae) was used to identify patients with C2 fracture in the NPR. ICD-10 has been validated for all diagnosis with an accuracy from 89 to 95% [20] and also for orthopaedic diagnosis with an accuracy of 95% for principal and secondary diagnoses until the third position and 90% to the fourth position [24]. The specificity of the ICD-10 code S12.1 has been validated in a dataset of 172 patients with ICD-10 S12.1 from 2002 to 2014, where 0% false positive cases were found (specificity = 100%). Baseline data for the included individuals were collected from the NPR and presented in tabular form. Causes of injury codes were not extracted from the NPR, due to, for our purposes, unacceptably low accuracy of these codes [20, 24]. Patients receiving surgical treatment were identified, using Swedish surgical procedure codes for spinal fusion (“NAG”) and spinal fracture treatment (“NAJ”). Nonsurgically treated cases with a change of treatment modality to surgery were registered as surgical patients. Subgroup analysis was performed for nongeriatric (20–69 years) and geriatric patients (70–99 years) and for nonsurgical and surgical treatment.

### 2.5. Statistical Methods

All statistical calculations were programmed in R version 3.3.0 (R Foundation for Statistical Computing, Vienna, Austria) [25]. Mean values were presented ± standard deviation if not indicated otherwise. Groups were compared with *t*-test for normally distributed variables; otherwise the Wilcoxon test was applied. Trends were analysed with linear regression and presented with correlation coefficient *r*. Group proportions were tested with *χ*^2^ test. *p* < 0.05 was regarded as statistically significant. The age distribution differences of patients with C2 fractures treated with and without surgery were visualised with a density distribution plot. A logistic regression analysis identified covariates of surgical treatment assignment and was presented with 95% confidence intervals (CI) and statistical probability *p* [26]. As relevant covariates in a model for surgical treatment assignment, age [27], gender [28], CCI [29], and SCI [30] were determined by literature review. Before removing the cases below 20 years of age and older 100 from the dataset, a histogram of the age-related frequency of C2 fractures was prepared.

### 2.6. Data Access and Cleaning Methods

The authors did not have direct access to the national registry databases in this study but were provided with a predefined extract from the national registries by the Swedish National Board of Health and Welfare (specification number: 13062/2015).

Even though a clean patient registry dataset was provided, duplicates (recurrent admissions of the same patient or continued treatment in a secondary facility) had to be identified and removed from the extract. The secondary diagnoses of the duplicates were added to the original record prior to duplicate exclusion.

## 3. Results

### 3.1. Participants

**Figure 1:**
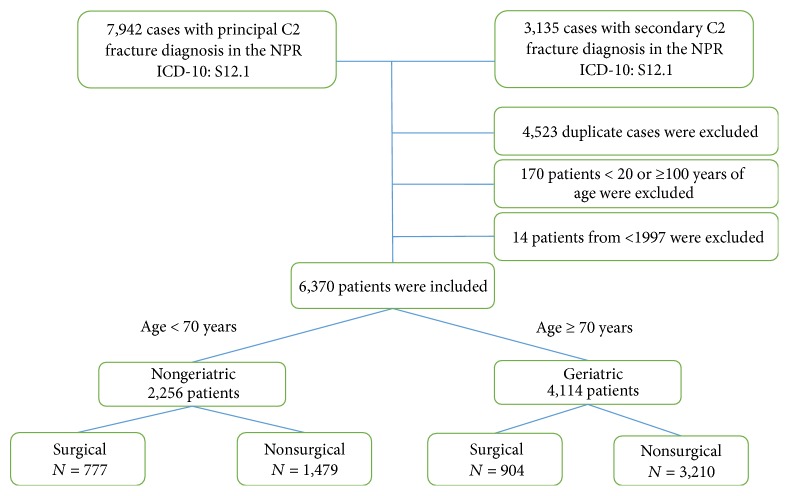
Inclusion flow diagram.

The population of 20 to 99 years of age in Sweden 1997 was 6,689,671 (mean age: 39.9 years), and in 2014 it was 7,536,133 inhabitants (mean age: 41.2 years). Between 1997 and 2014, a total number of 11,077 cases were treated as inpatients due to a C2 fracture. The inclusion flow chart is shown in Figure 1.

### 3.2. Descriptive Data

6,370 patients with the principal and secondary diagnosis of a C2 fracture (ICD-10: S12.1) were included. 51% were male. The mean age was 72 ± 18 years. The group was divided into nongeriatric patients < 70 years of age (*n* = 2,256) and geriatric patients ≥ 70 years of age (*n* = 4,114). 26% received surgical treatment: 34% in the nongeriatric group and 22% in the geriatric group (*χ*^2^ test, *p* < 0.01). Stratified for gender (51% male, 49% female), 31% male and 22% female patients received surgical treatment (*χ*^2^, *p* < 0.01).

Baseline data is shown in Table 1. The Charlson Comorbidity Index (CCI) was 4.9 ± 2.5, and spinal cord injury (SCI) was present in 2% (*n* = 140). 10% (*n* = 630) had a concomitant C1 fracture.

### 3.3. Outcome Data 

#### 3.3.1. Incidence of C2 Fractures

**Figure 2:**
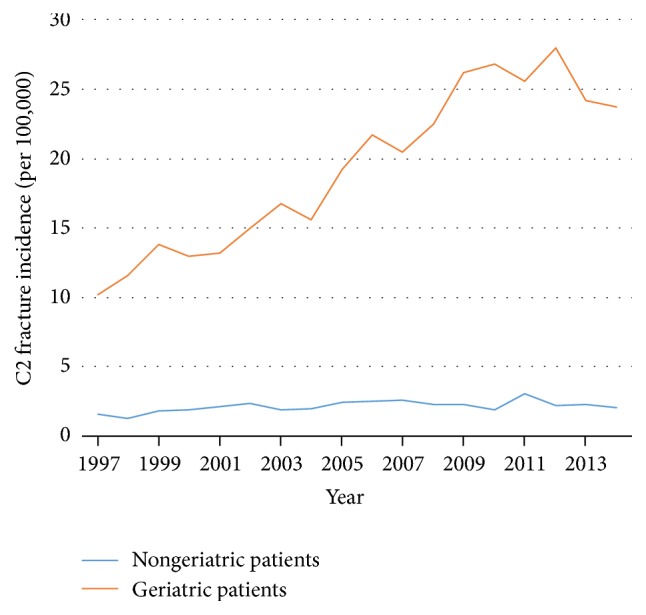
C2 fracture incidence per 100,000 of nongeriatric (blue) and geriatric (red) patients between 1997 and 2014.

The incidence of C2 fractures doubled from 1997 to 2014 from 3 to 6 per 100,000 inhabitants (*r* = 0.94; *p* < 0.01). The incidence in the geriatric group increased linearly from 10.2 to 23.7 per 100,000 from 1997 to 2014, which was not found in the nongeriatric group (Figure 2) (*r* = 0.89; *p* < 0.01).

**Figure 3:**
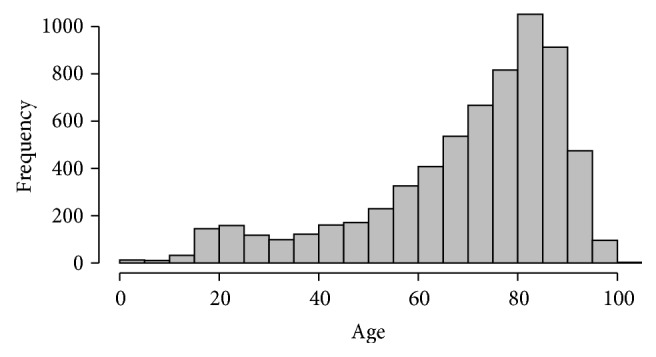
Age distribution of C2 fractures.

**Figure 4:**
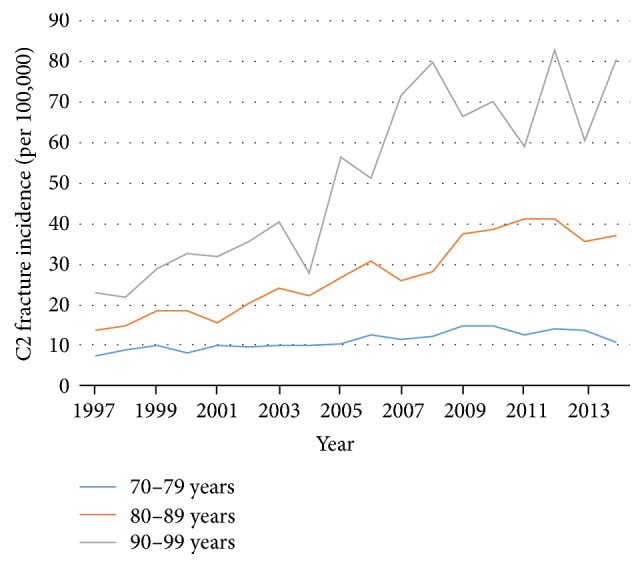
Incidence of C2 fractures between 1997 and 2014 per 100,000 in geriatric age categories: 70–79 years (blue), 80–89 years (orange), and 90–99 years (grey).

There was no significant difference of the C2 fracture incidence between the sexes in the subgroup of 80–89 and 90–99 years of age (*p* = 0.43 and *p* = 0.46). With regard to patients below the age of 80 years, C2 fractures were more common in men (*p* < 0.01) (Table 2). A bimodal distribution of age-related C2 fracture frequency was found with peaks at 20–25 years and at 80–85 years (Figure 3). From 1997 to 2014, the C2 fracture incidence quadrupled in the old geriatric patients (90–99 years), while it more than doubled in the age group of 80–89 years and it increased by 30% in the age group of 70–79 years (Figure 4).

#### 3.3.2. C2 Fracture Treatment Trends in Sweden

**Figure 5:**
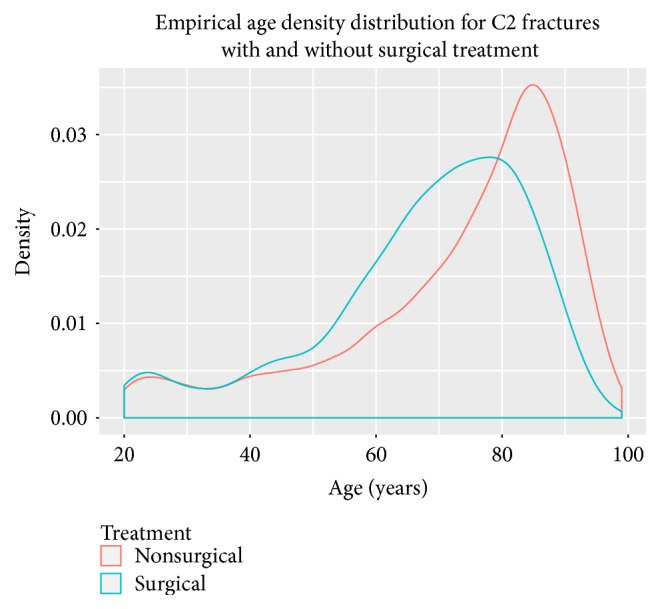
Empirical age distribution for C2 fractures with and without surgical treatment.

**Figure 6:**
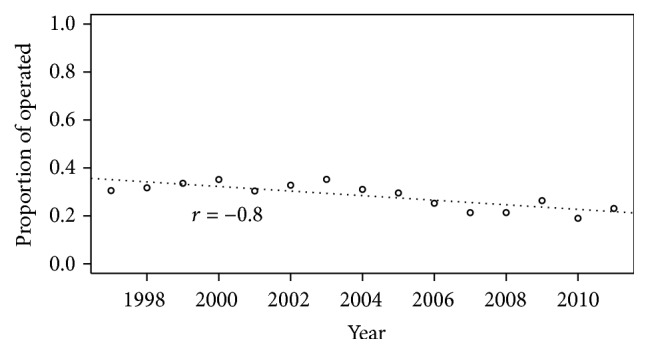
Annual proportion of surgically treated patients with C2 fractures.

**Figure 7:**
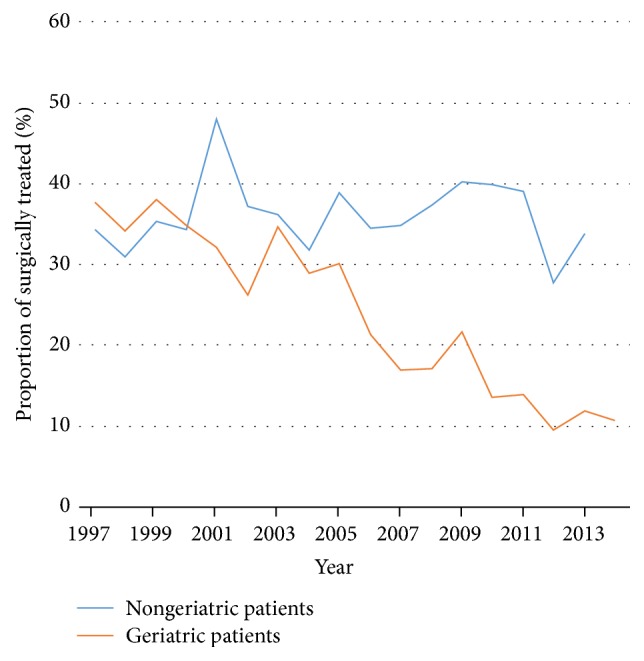
Proportion of surgically treated nongeriatric (blue) and geriatric (orange) patients with C2 fractures between 1997 and 2014.

Of the included patients, 26% were treated surgically. There was a higher density of nonsurgical treatment in the elderly (Figure 5). There has been linear trend from 1997 to 2014 towards nonsurgical treatment (*r* = −0.8; *p* < 0.01) (Figure 6). There was an even stronger trend towards nonsurgical treatment in the geriatric subgroup (*r* = −0.95; *p* < 0.01), compared to the younger age group. Treatment trends are shown in Figures 5, 6, and 7.

In a logistic regression model, the odds ratio of surgical treatment assignment was significantly greater for younger age, male gender, SCI, and earlier year of admission (Table 3).

## 4. Discussion

### 4.1. Key Results

This study documents a growing incidence and a declining surgical treatment trend of C2 fractures in the elderly during the last two decades in Sweden.

### 4.2. Interpretation

#### 4.2.1. Incidence of C2 Fractures

Since 1997, the incidence of C2 fractures has risen from 3 to 6 per 100,000. As the elderly population has grown dramatically in Sweden, the number of hospital admissions due to elderly-specific C2 fractures increased during the last decade. Despite the 64% increase in the population of 90 to 99 years of age from 1997 to 2014, a 4-fold increase of the population-adjusted incidence of C2 fractures was found. This compares to the 70–79 years of age population which only increased by 13% but C2 fractures increased by 43%.

One explanation for the increased incidence of C2 fractures is a diagnostic bias, as we nowadays use a computed tomography instead of conventional radiographs as a first diagnostic instrument [31]. Beyond that, the number of falls in the elderly is substantial [32]. There is an increased rate of falls, 78%, for those with four or more risk factors [33, 34]. 5% of the falls cause a fracture [34]. The elderly receive better treatment for comorbidities compared to decades ago [35]. This leads to a higher activity level of the elderly, along with a higher risk of falling, but also the inactive persons stand a high risk of falls [32]. The orthostatic effect of medication like benzodiazepines and antihypertensive drugs may also lead to falls. Furthermore, the fact that the healthcare system in Sweden encourages geriatric patients to live in their own homes instead of nursing homes affects possibilities of supervision and accessibility, a plausible cause of domestic falls [35, 36]. Otherwise, patients at nursing homes stand a higher risk of falls [32, 34]. The combination of falls, a stiff lower cervical spine, and osteoporosis could explain the increased incidence of C2 fractures in the elderly [37, 38].

#### 4.2.2. C2 Fracture Treatment Trends in Sweden

There was a national trend towards nonsurgical treatment of C2 fractures in Sweden, foremost in the elderly, which does not confirm previously published results from other countries [37, 39, 40]. Fear of overtreatment could be a factor contributing to the trend of nonsurgical treatment of cervical fractures. The elderly patients' comorbidity could explain the physicians' tendency to use a cervical collar in the belief of avoiding harm. In contrast, recently published results suggest that surgical C2 fracture stabilisation reduces morbidity and mortality of elderly patients with greater comorbidity [37, 41]. In this registry study, we could not perform a subgroup analysis of C2 fracture subtypes, level of dislocation, or treatment allocation. Several authors recommend a treatment based on level of fracture dislocation besides comorbidity and age; this could not be investigated in our cohort [2, 6, 42].

#### 4.2.3. Gender Differences in Treatment Assignment

This study pinpointed that men and women were treated differently. The proportion of women treated surgically was much lower than men. Thus, female patients received a probably inferior treatment with regard to survival [43]. Multiple studies have documented an implicit, unintended discrimination of female patients by their physicians [44, 45]. As treating surgeons, we should accept and acknowledge the fact that our treatment decisions are unintentionally affected by stereotypes as gender [40]. This will allow us to minimise implicit gender discrimination.

### 4.3. Strengths and Limitations

Due to the unmatched coverage and the high internal validity of the Swedish patient registry, the presented data is reliable. The national population-based cohort design of this study minimises the selection bias of tertiary referral centres. These often attract odd and unusual case referrals and distort the disease panorama. This national registry study has therefore advantages over many previously published cohort studies. Furthermore, registry studies have the strength of including the whole population instead of creating a sample of the population (as you would do in randomised controlled trials) [37]. This allows identification of even rare diseases or complications of treatments.

As the ICD-10 does not allow the differentiation of odontoid fractures from other C2 fractures, the NPR could not answer the question of proportion of C2 fracture subtypes. In a previous study from two regions in Sweden, we have revealed that about 63% of the C2 fractures are odontoid type 2 fractures in the elderly population ≥ 70 years, and 26% are odontoid type 3 fractures [8]; this means that a total 89% of the odontoid fractures in the elderly are either type 2 or type 3. We described an increase in the proportion of odontoid C2 fractures in the elderly from 2002 to 2014. Therefore, one can assume that the increase of C2 fractures in the geriatric subgroup from 1997 to 2014 found in the present study was largely due to an increase of odontoid type 2 and type 3 fractures. In the younger age group, our previous study from Sweden revealed a more differentiated panorama of C2 fractures, including 24% Hangman's fractures, 21% atypical fractures, 17% odontoid type 3 fractures, and 34% odontoid type 2 fractures [8]. In the present nationwide study, C2 fractures of the nongeriatric patients did not increase (Figure 2); thus, any conclusions regarding the C2 fracture subtype distribution would be speculative.

The availability of computed tomography for diagnostics of cervical injuries in the last two decades could have led to a diagnostic bias, where a greater number of C2 fractures would be detected during the recent years of this study [46, 47].

As the validity of the 4th digit of the fracture ICD-10 code (90%) is lower than the third digit (95%) [24], approximately 5% of C2 fractures were likely to be misdiagnosed as other cervical spine fractures (S12.0, S12.2, S12.7, S12.8, and S12.9). In contrast, the risk that fractures that are not C2 fractures were misdiagnosed as S12.1 is low, since the specificity of S12.1 was 100% (unpublished data).

A confounder not controlled for in this study is the comorbidity of osteoporosis. If the population's osteoporosis improved (i.e., due to better preventive healthcare measures), this would affect the risk of cervical spinal fractures [35].

As most other countries, Sweden has a geographically, health-economically, and ethnically unique population. The results presented in this study might not be generalizable to the rest of the world. Studies from national patient registries in other countries will have to validate our results in their specific settings.

## 5. Conclusion

This study identified an increased incidence of C2 fractures during the last decade along with a decreased proportion of surgically treated elderly patients. Results from ongoing randomised controlled trials, as the U-SOFT trial (ClinicalTrials.gov # NCT02789774), will facilitate an evidence-based treatment rationale for C2 fractures.

## Figures and Tables

**Table 1:** Baseline values of patients according to treatment presented as count (*n*) or mean ± standard deviation.

	*n*		Sex		CCI		Spinal fracture				Surgical technique	
		Age	Male	Female		SCI	C1	Subaxial	T	L	Screw	Fusion
		Years	*n*	*n*		*n*	*n*	*n*	*n*	*n*	*n*	*n*
Surgical	1681	68 ± 17	1013	668	4.4 ± 2.3	77	236	194	90	30	168	1513
Nonsurgical	4689	73 ± 18	2277	2412	5.1 ± 2.6	63	394	272	186	122	0	0

All	6370	72 ± 18	3290	3080	4.9 ± 2.5	140	630	466	276	152	168	1513

*n*: number; SD: standard deviation; CCI: Charlson Comorbidity Index; SCI: spinal cord injury; T: thoracic; L: lumbar.

**Table 2:** Incidence of C2 fractures per 100,000 within age subgroups according to gender (presented with *p* values of *t*-test for gender difference).

Age category Years	Female Per 100,000	Male Per 100,000	Both sexes Per 100,000	*t*-test *p* value
20–29	0.8	2.1	1.5	<0.01
30–39	0.5	1.4	1.0	<0.01
40–49	0.8	2.1	1.4	<0.01
50–59	1.7	3.0	2.4	<0.01
60–69	3.9	5.9	4.9	<0.01
70–79	9.2	13.6	11.2	<0.01
80–89	26.2	28.8	27.2	0.43
90–99	49.7	55.2	51.1	0.46

**Table 3:** The assignment to surgical treatment was dependent on younger age, male gender, spinal cord injury, and earlier year of admission. Odds ratios are presented with 95% CI and *p* value.

	OR	95% CI		*p*
		2.5%	97.5%	
Age	0.99	0.99	1.00	0.012
Male gender	1.42	1.26	1.59	<0.001
Spinal cord injury	2.94	2.08	4.16	<0.001
Charlson Comorbidity Index	0.96	0.92	1.00	0.072
Year of admission	0.96	0.94	0.97	<0.001
